# 肺癌根治术中胸膜腔冲洗液肿瘤细胞检查的研究进展

**DOI:** 10.3779/j.issn.1009-3419.2018.09.12

**Published:** 2018-09-20

**Authors:** 东来 陈, 冉冉 付, 平凡 石, 塽 覃, 昶 陈

**Affiliations:** 1 200433 上海，同济大学附属上海市肺科医院 Department of Thoracic Surgery, Shanghai Pulmonary Hospital, Tongji University School of Medicine, Shanghai 200433, China; 2 200092 上海，同济大学医学院 Tongji University School of Medicine, Shanghai 200092, China

**Keywords:** 肺肿瘤, 术中胸腔冲洗液, 肿瘤细胞, 预后, Lung neoplasms, Intraoperative pleural irrigating fluid, Tumor cells, Prognosis

## Abstract

非小细胞肺癌（non-small cell lung cancer, NSCLC）根治术中胸腔冲洗液细胞学检查是一种常用的检测肿瘤进展并评估患者预后的诊断技术。长期以来大量研究者致力于阐明术中胸腔冲洗液细胞学检查结果与肺癌患者术后生存及肿瘤复发转移的关系。由于不同研究间存在明显异质性，其结论也各有不同。但能肯定的是，胸腔冲洗液肿瘤细胞检查阳性已被证实为手术患者的不良预后因素之一。本文尝试从临床意义、影响因素及可能发生机制等角度就近年来术中胸膜腔冲洗的研究进展作一综述。

## Abstract (en)

Intraoperative pleural lavage cytology is a diagnostic technique used to detect tumor cells and serve as a prognostic parameter for non-small cell lung cancer (NSCLC) patients. In the past several decades, many scholars have been dedicated to clarifying the relationships between positive intraoperative pleural lavage cytology results and postoperative survival as well as tumor recurrence and metastasis. However, the findings remained various due to the inhomogeneity of different research. It has been confirmed that a positive intraoperative pleural lavage cytology result is one of the risk factors for the prognosis of postoperative patients. This study reviewed the advances in research of intraoperative pleural lavage cytology in recent years from several aspects, including clinical significance, influencing factors and possible mechanisms.

随着2017年1月第8版肺癌肿瘤-淋巴结-转移（tumor-node-metastasis, TNM）分期的实施，肺癌根治术前被发现存在恶性胸水的患者被列为M1a期，往往丧失了手术机会^[1]^。而长期以来，虽然有大量研究聚焦于肺癌根治术中胸膜腔冲洗液内肿瘤细胞的检测^[2-6]^，并已证实无论肺切除前或切除后的冲洗液中检出肿瘤细胞均与预后有关^[7, 8]^，但迄今胸膜腔冲洗液中肿瘤细胞的存在仍未被列为TNM分期的参考指标，其预后意义也众说纷纭。近年来，亦有学者开始关注胸膜腔冲洗液中肿瘤相关指标与预后的关系^[9, 10]^。此外，目前就使用何种液体进行术中胸膜腔冲洗尚未达成专家共识，相关研究也鲜有报道。本文就近年来术中胸膜腔冲洗液肿瘤细胞检查的研究进展作一综述。

## 1 胸膜腔冲洗液细胞学检查

胸膜腔冲洗液细胞学检查（pleural lavage cytology, PLC）是一种用于检测肿瘤细胞亚临床播散的技术，其结果不作为影响手术切除决策的依据^[11, 12]^。目前，学界尚无明确标准将肿瘤细胞胸膜播散和PLC阳性结果进行区分，但既往研究均指出PLC阳性与预后不良相关。

在当前已发表的临床研究中，研究者们对术中行PLC的时机进行了分类，大致可分为：①进胸后行肺切除前行PLC；②肺切除并行淋巴结清扫后立刻行PLC；③关胸前行PLC^[8]^三类。亦有研究者将其分为：①肺切除前行PLC；②肺切除后行PLC^[7]^或①进胸后立刻行PLC；②关胸前行PLC^[13]^。在肺癌根治术中，进胸后肺切除前的PLC检测结果阳性往往提示肿瘤细胞的早期播散^[6]^，肺切除后关胸前的PLC检测结果相比肺切除前行PLC不仅具有更大的预后价值^[7, 13, 14]^，而且可以作为指导术后辅助治疗的参考^[8, 15]^。尽管目前学界对PLC能否纳入TNM分期尚存争议^[11]^，然而其检测结果对接受肺癌根治术患者的预后分层作用及治疗指导意义已愈发受到临床医生的认可与重视。

2010年国际PLC协作组对先前来自22个医学中心发表的31篇临床研究进行了荟萃分析，结果提示PLC可被推荐为非小细胞肺癌（non-small cell lung cancer, NSCLC）手术患者的常规检查项目，其阳性结果为患者术后生存的独立风险因素，且PLC阳性的Ⅰ期-Ⅲ期NSCLC患者T分期应上升一个等级^[16]^。2014年又一项基于日本肺癌登记数据库的研究^[17]^在纳入4, 171例患者进行回顾性分析后，亦给出了相同的结论。遗憾地是，由于上述荟萃分析的文献异质性及研究的回顾性特质，各研究团队纳入的患者在术中行PLC的时机可能存在差异，因此与术后病理分期升级关系最密切的PLC结果不得而知。

## 2 PLC阳性结果的影响因素

在不同研究及同一研究的不同患者群组中，PLC的阳性检出率差异极大，大致在3.7%-38.6%之间^[11]^，但上述相去甚远的阳性检出率既可归因于肿瘤自身属性如大小、病理类型、侵袭性等；亦与行PLC的时机及获取样本的方法有关^[11]^。在肺切除前PLC阳性检出率差异可达2.7%-22.6%，切除后亦可达3.7%-13.5%；而冲洗时使用的生理盐水量在不同研究中差异可达20 mL-2, 000 mL不等^[7, 8, 12, 18]^，在冲洗液标本的具体检测方法和阳性判断标准上也可存在差异。近十年来多数研究^[8, 12-15, 19-21]^均采取Papanicolaou方法进行PLC的判定，根据Papanicolaou的结果可将冲洗液标本分为5个等级：Ⅰ-Ⅲ为阴性，Ⅳ-Ⅴ为阳性。

### 2.1 肺切除前PLC阳性

表 1总结了近十年来文献中分析得出的与肺切除前PLC阳性相关的因素。从中我们不难看出肺切除前PLC阳性率与肿瘤的病理直径（T分期）、胸膜浸润情况、淋巴结累及情况（N分期）和病理分期（P分期）高度相关，且可能与病理类型、脉管侵犯情况相关，甚至可能与患者年龄相关。同时亦有文献报道肺切除前PLC阳性率或与性别、术前CEA水平相关^[22]^。此外，连同表 1内所列的多项研究^[8, 13, 19, 20, 22, 23]^均一致指出术前PLC可作为NSCLC手术患者的独立预后因素。

**1:** 肺切除前胸腔冲洗液细胞学检查阳性结果的相关因素 Factors associated with positive pre-resectional PLC

Author, ref.number, year, type of study	Patient group and saline volume for washing	Factors associated with positive pre-resectional PLC (*P* value)
Shintani *et al*^[8]^ (2008) Prospective study	Total number of patients: 1, 271 Patients with positive PLC before lung resection: 67 (5.3%) 500 mL physiologic saline solution	Pathologic stage (*P*=0.039, 7) Pathologic pleural involvement (*P*＜0.000, 1) Pathologic T status (*P*=0.000, 2) Vascular invasion (*P*=0.004, 8)
Taniguchi *et al*^[13]^ (2009) Prospective study	Total number of patients: 296 Patients with positive PLC after thoracotomy: 14 (4.7%) 50 mL warm physiologic saline solution	T stage (*P*=0.028) Pleural invasion (*P*＜0.000, 1) Lymphatic invasion (*P*=0.002) N stage (*P*=0.369) Vascular invasion (*P*=0.176)
Kaneda *et al*^[18]^ (2011) Retrospective study	Total number of patients: 3, 231 Patients with positive PLC immediately after thoracotomy: 148 (4.58%) Physiological saline of ≤100 mL	Histology (adenocarcinoma) (*P*=0.015) Pathological stage (*P* < 0.001) Pathological T score (*P* < 0.001) Pathological N score (*P* < 0.001) Pleural invasion (*P* < 0.001)
Yanagawa *et al*^[19]^ (2014) Prospective study	Total number of patients: 428 Patients with positive PLC immediately after thoracotomy: 19 (4.4%) 50 ml physiological saline solution	Tumor size (*P*=0.008) Pathological stage (*P* < 0.001) Pleural invasion (*P* < 0.001)
Kameyama *et al*^[17]^ (2014) Retrospective study	Total number of patients: 4, 171 Patients with positive PLC immediately after thoracotomy: 217 (5.2%) Volume unknown	Histology (adenocarcinoma) (*P*＜0.000, 1) Pathological T category (*P*＜0.000, 1) Pathological N category (*P*＜0.000, 1) Pathological M category (*P*＜0.000, 1) Pathological stage (*P*＜0.000, 1) Pleural factor (*P*＜0.000, 1)
Nakao *et al*^[12]^ (2015) Retrospective study	Total number of patients: 1, 572 Patients with positive PLC immediately after thoracotomy: 56 (3.6%) 100 mL physiological saline solution	Pathological T classification (*P* < 0.001) Pathological N classification (*P* < 0.001) Pleural invasion (*P* < 0.001)
Tomizawa *et al*^[20]^ (2016) Retrospective study	Total number of patients: 860 Patients with positive PLC immediately after thoracotomy: 38 (4.4%) 50 mL physiological saline solution	Age (*P*=0.002) Pleural invasion (*P*＜0.000, 1)
PLC: pleural lavage cytology.		

关于肺切除前PLC阳性检出率与NSCLC病理亚型的相关性，目前有两种不同论调：一种观点认为肺切除前PLC阳性更多见于腺癌这一病理类型^[6, 22, 24]^，并归因于肿瘤本身的高侵袭性；另一种观点则认为鳞癌更易出现肺切除前PLC阳性^[25, 26]^，但亦有人指出鳞癌患者的阳性结果或由冲洗液中呈团簇状的间皮细胞与肿瘤细胞混淆而引起^[27]^，即存在假阳性。Li等^[28]^的荟萃分析则指出病理类型与肺切除前PLC阳性结果之间无明确相关性。

肺切除前PLC阳性与胸膜浸润的相关性已见诸表 1中多篇文献。肺切除前PLC常见于胸膜浸润分级为p2的患者中（肿瘤细胞侵犯至脏层胸膜表面）^[29, 30]^，这或与冲洗过程中液体的机械力将肿瘤细胞从脏层胸膜剥离有关，且可能造成人为的肿瘤细胞胸膜腔播散；而在p0（肿瘤细胞未侵及脏层胸膜弹力纤维层）和p1（肿瘤细胞突破脏层胸膜弹力纤维层）患者中亦有肺切除前PLC阳性报道^[4, 6, 27]^；但p3患者（肿瘤细胞侵犯至壁层胸膜）中此类报道则并不常见。亦有研究^[25]^认为肺切除前PLC阳性与脏层胸膜浸润并无明确相关性，而与肿瘤累及胸壁密切相关（T3分期肿瘤）。

### 2.2 肺切除后PLC阳性

表 2总结了近十年来文献中所列的与肺切除后PLC阳性相关的因素，如表 2所示，肺切除后冲洗液用量显著高于切除前，而500 mL以上的用量可造成结果假阴性^[18]^。迄今为止，多项研究发现肺切除后PLC阳性与N分期、胸膜浸润情况、淋巴管及微血管侵犯以及病理分期有关^[7, 8, 13, 31]^，但亦有不同观点认为肺切除后PLC阳性与TNM分期、胸膜浸润和淋巴管侵犯无关^[24, 32]^。此外，Dresler等提出肺切除后PLC阳性可能与肿瘤分化程度相关，而Enatsu等^[7]^的一项大规模前瞻性研究发现肺切除后PLC阳性与手术切除彻底性（无切缘阳性、进行系统性淋巴结清扫为彻底切除）成负相关。另一不容忽视的影响因素是手术操作本身亦可引起肺切除后PLC阳性，且术前经胸壁穿刺活检^[11]^或行结节穿刺定位亦为造成肿瘤细胞脱落至胸膜腔内的危险因素。若研究者同时纳入肺切除前后PLC结果^[7, 13]^，则研究流程本身就使肺切除后的PLC结果不可避免地受切除前冲洗的影响。

**2:** 肺切除后胸腔冲洗液细胞学检查阳性结果的相关因素 Factors associated with positive post-resectional PLC

Author, ref. number, year, type of study	Patient group and saline volume for washing	Factors associated with positive pre-resectional PLC (*P* value)
Shintani *et al*^[8]^ (2008) Prospective study	Total number of patients: 1, 271 Patients with positive PLC after lung resection: 32 (2.5%) 500 mL physiologic saline solution	Pathologic T status (*P*=0.001, 6) Vascular invasion (*P*=0.046, 8)
Taniguchi *et al*^[13]^ (2009) Prospective study	Total number of patients: 296 Patients with positive PLC before closure: 26 (8.8%) 2, 000 mL warm physiologic saline solution	N stage (*P*＜0.000, 1) P factor (*P*=0.002) Lymphatic invasion (*P*＜0.000, 1) Vascular invasion (*P*＜0.000, 1)
Nakao *et al*^[12]^ (2015) Retrospective study	Total number of patients: 1, 135 Patients with positive PLC before closure: 3 (0.26%) 2, 000 mL-3, 000 mL warm physiologic saline solution	Excluded from subsequent analyses due to lack of data

## 3 PLC阳性的可能机制

目前恶性胸水形成的病理生理机制被总结为肺内淋巴管的损伤及阻塞导致体液引流不畅形成的漏出液积聚，而PLC阳性不伴有恶性胸水的相关机制则尚待进一步阐明。目前主流观点认为PLC阳性是由部分侵袭性高的肿瘤细胞经胸膜下淋巴管或受累纵隔淋巴结穿透至胸膜腔内引起的，或先到达胸膜表面再被人为操作剥落至冲洗液中^[4, 6, 11, 27, 32]^。这种解释符合上述PLC阳性与脉管侵犯相关的病理发现，PLC阳性因此也被视为隐匿性的胸膜播散^[11]^。亦有观点认为肿瘤细胞可与被吸入的细颗粒物一样经外周肺实质进入胸膜腔^[33]^。而上文提到的p3患者中PLC阳性与壁层胸膜累及相关性的机制当下仍存在争议，有观点认为肿瘤细胞与壁层胸膜及胸壁之间可形成纤维粘附^[27]^，阻止肿瘤细胞在胸膜腔内播散因而不易出现PLC阳性结果。此外，由穿刺针道引起的胸膜腔内人为播散亦被视为引起PLC阳性的可能机制^[34]^，但亦有研究提出二者之间无明确相关性^[5, 24, 35]^。

肺切除前PLC阳性往往与肿瘤的自然史相关，是肿瘤侵袭力增强、疾病进展的体现；而肺切除后PLC阳性则被认为与手术操作及冲洗液的机械性作用相关性更大^[11]^，且手术本身在清扫纵隔淋巴结的同时可能破坏被肿瘤细胞侵犯的淋巴管及小血管，造成脉管中肿瘤细胞被人为释放到冲洗液中^[34, 36]^。尽管这种人为造成肿瘤胸膜腔或胸壁播散的风险可被多次大量冲洗降至最低，但极易引起切除后PLC的阳性发现。总之，当前关于PLC阳性的病理生理机制尚缺乏统一的认识。

## 4 PLC阳性的临床意义

表 3汇总了近十年来探究肺切除前PLC与预后关系的研究及其结论。由表 3可见肺切除前PLC阳性患者术后生存状况显著差于阴性患者，但其是否可作为独立预后因素尚存争议。一些研究还发现肺切除前PLC阳性对预后的不利影响在早期肺癌患者中更为显著，而在晚期患者中则并不明显，这或与晚期患者本身预后极差有关^[24, 29]^。具体表现为其对Ⅰ期和Ⅱ期肺癌患者术后生存期的预测价值^[4, 22, 24, 26]^，且已有日本的多中心大规模回顾性研究作为支撑和验证^[37]^，该项研究亦提出肺切除前的PLC阳性结果仅对Ⅰ期和Ⅱ期患者预后产生不利影响，而对Ⅲa期患者预后影响甚微。因此当前多数学者倾向于将肺切除前PLC阳性的T分期升级到T3^[18, 20]^或T4^[14, 16, 17]^，以示与恶性胸水形成的Ⅳ期（M1a）患者的预后进行区分。同样，在Ⅰ期和Ⅱ期肺癌患者中，肺切除前PLC阳性者较阴性者更易出现局部和远处转移^[22, 35]^；而这种区分度在Ⅲa期和Ⅲb期患者中亦明显缩小^[11]^。此外，肺切除前PLC阳性的早期肺癌患者术后更易出现以癌性胸膜炎为形式的疾病复发^[24, 37]^，这也部分解释了某些早期患者在接受根治性切除后短时间内出现局部复发的现象。Ichinose等^[38]^的一项前瞻性研究则提出，针对肺切除前PLC阳性患者使用低渗性铂类冲洗液相比常规的生理盐水冲洗，可显著降低患者术后癌性胸膜炎的发生率，但因入组过慢致该研究提前终止，最终仅纳入49例，存在样本量过小且未体现术后生存优势的问题。此外，Ogawa等^[15]^于2015年发表的一项回顾性研究表明，对包括病理Ⅰ期在内的术中PLC阳性患者，手术联合术后辅助化疗相比单纯手术可显著改善患者术后的无复发生存期，而在该项研究中，行PLC的时机为进胸后立即送检，因此该项研究有着明显时机局限性，证据等级亦尚显不足。

**3:** 探究开胸后肺切除前行胸腔冲洗液细胞学检查预后意义的相关研究 Studies demonstrating the prognostic significance of pleural lavage cytology immediately after thoracotomy and before lung resection

Author, ref. number, year, type of study	Patient group	Outcomes: survival rate	Other results or comments
Shintani *et al*^[8]^ (2009) Prospective study	Total number of patients: 1, 271 Patients with positive PLC before lung resection: 67 (5.3%)	Five-year survival in pre-PLC positive patients was 44.1% compared to 58.3% in pre-PLC negative patients (*P*=0.039, 4)	PLC before lung resection cannot be used as an independent prognostic factor.
Taniguchi *et al*^[13]^ (2009) Prospective study	Total number of patients: 296 Patients with positive PLC after thoracotomy: 14 (4.7%)	5-year survival rate for negative PLC vs positive PLC after thoracotomy: 72% vs 45% (*P*=0.047)	A significant correlation was found between PLC after thoracotomy and T factor.PLC after thoracotomy is not an independent prognostic factor.
Kawachi *et al*^[21]^ (2009) Retrospective study	Total number of patients: 563 Patients with positive pre-PLC: 41 (7.2%)	Patients positive for pre-PLC had significantly worse survival than pre-PLC negative patients (*P*＜0.000, 1)	The incidence of intrathoracic recurrence was significantly higher in pre-PLC positive patients. Positive pre-PLC was found to be an independent prognostic factor.
Aokage *et al*^[14]^ (2010) Prospective study	Total number of patients: 2, 178 Patients with positive pre-PLC: 65 (3.0%)	The 5-year survival rate was 37% in 65 patients without dissemination but with a positive pre-PLC result, which was significantly higher than 12% in 86 patients with dissemination (*P*=0.002)	Pre-PLC proved to be a strong independent prognostic factor, but was of less use in clinical practice compared with post-PLC.
Kaneda *et al*^[18]^ (2012) Retrospective study	Total number of patients: 3, 231 Patients with positive pre-PLC: 148 (4.58%)	Survival curves were significantly worse (*P* < 0.001) in the pre-PLC positive group	A positive pre-PLC result was found to be a poor prognostic indicator. The incidence of recurrence with pleuritis carcinomatosa was significantly higher in the pre-PLC positive group.
Nakao *et al*^[12]^ (2015) Retrospective study	Total number of patients:1, 572 Patients with positive pre-PLC: 56 (3.6%)	Recurrence was observed more frequently (*P* < 0.001) and the rate of pleural recurrence at the initial site was higher (*P* < 0.001) in pre-PLC positive patients.	Positive pre-PLC had the significant prognostic effect in surgically resected NSCLC patients. However, it is not a contraindication for surgical resection and should be classified as pT3.
Tomizawa *et al*^[20]^ (2016) Retrospective study	Total number of patients: 754 Positive pre-PLC immediately after thoracotomy: 38 (5.1%)	The 5-year OS of patients with positive PLC was significantly shorter than that of those with negative PLC and pT1 (*P*＜0.000, 1) or negative PLC and pT2 (*P*＜0.000, 1) and almost overlapped with that of those with negative PLC and pT3 disease (*P*=0.601)	Positive PLC was an independent prognostic factor in patients with resected NSCLC. Patients with positive PLC should be staged as pT3.
Shoji *et al*^[34]^ (2016) Prospective study	Total number of patients: 700 Positive pre-PLC immediately after thoracotomy: 58 (8.2%)	The 5-year OS of patients with positive pre-PLC was significantly worse than those with negative pre-PLC (*P*=0.000, 1). The RFS was similarly better for patients with negative post-PLC status regardless of pre-PLC findings (not significant).	Post-PLC status but not pre-PLC status together with pathologic N factor and pathologic stage, was identified as an independent factor for poor prognosis (*P*=0.004, 0).
pre-PLC: pleural lavage cytology before lung resection or immediately after thoracotomy; post-PLC: pleural lavage cytology after lung resection; OS: overall survival; RFS: Recurrence-free survival.			

如表 4所示，肺切除后PLC阳性不仅是患者术后生存的不利因素，而且已有多项研究经多因素分析提出其相比切除前PLC阳性具有更大的预后价值^[7, 14, 34]^。甚至有研究提出肺切除后PLC阳性可成为等价于恶性胸腔积液的独立预后因素^[14, 32]^。同时肺切除后PLC阳性与术后局部复发及远处转移的相关性也更显著，但不可否认的是胸腔内局部复发亦可能由关胸前冲洗残留的肿瘤细胞所致，至于其导致远处转移的具体机制则不得而知。总体而言，近年来关于切除前PLC阳性预后意义的研究数量远多于切除后PLC阳性，这或与学界已逐步达成对肺切除后PLC阳性的一致看法有关，即承认肺切除后PLC阳性是预后的独立危险因素。

**4:** 探究肺切除后行胸腔冲洗液细胞学检查预后意义的相关研究 Studies demonstrating the prognostic significance of pleural lavage cytology after lung resection

Author, ref. number, year, type of study	Patient group	Outcomes: survival rate	Other results or comments
Taniguchi *et al*^[13]^ (2009) Prospective study	Total number of patients: 296 Patients with positive PLC after lung resection: 26 (8.8%)	5-year survival rate for negative PLC vs positive PLC before closure: 76% vs 32% (*P*＜0.000, 1)	PLC before closure was found to be an independent prognostic factor (*P*=0.001) in multivariate analysis, in contrast with PLC after thoracotomy (*P*=0.26)
Shintani *et al*^[8]^ (2009) Prospective study	Total number of patients: 1271 Patients with positive post-PLC: 32 (2.5%)	Five-year survival rate was 21.9% for patients with positive post-PLC compared to 58.3% for patients with negative post-PLC (*P*＜0.000, 1)	A significantly higher rate of pleural recurrence was found in patients with positive post-PLC compared to patients with negative post-PLC
Aokage *et al*^[14]^ (2010) Prospective study	Total number of patients: 2178 Patients with positive post-PLC: 70 (3.2%)	The 5-year survival rate for positive post-PLC vs negative post-PLC: 22% vs 69% (*P* < 0.001). The5-year recurrence-free survival rate for positive post-PLC vs negative post-PLC: 4% vs 67% for patients with (*P* < 0.001).	Post-PLC showed a stronger survival impact than pre-PLC. Post-PLC was also a strong predictor of recurrence.
Shoji *et al*^[34]^ (2016) Prospective study	Total number of patients: 700 Patients with positive post-PLC: 47 (6.7%)	The 5-year OS of patients with positive post-PLC was significantly worse than those with negative pre-PLC (*P*＜0.000, 1). The RFS was similarly worse for patients with positive post-PLC status regardless of pre-PLC findings (not significant).	Differences between the RFS according to post-PLC status were greater than those according to the pre-PLC status. Multivariate analysis of RFS revealed that post-PLC status (*P*=0.009, 0) is an independent prognostic factor.

## 5 PLC与其他肿瘤相关指标

近年来，学者们在关注术中冲洗液内的肿瘤细胞之外，也逐步开始探索PLC阳性与冲洗液中其他指标的关系。Inoue等^[10]^指出，EGFR外显子21 L858R存在突变的患者相比没有*EGFR*突变的患者更容易出现肺切除前PLC阳性结果。这或对部分EGFR突变的肺癌患者预后评估及后续治疗指导起到启示作用。Tsutani等^[9]^发现术中冲洗液CEA水平与肿瘤大小、胸膜浸润及血清CEA水平相关，而与肺切除前PLC无关，但冲洗液中CEA相比血清CEA具有更显著的预后意义。目前术中冲洗液内肿瘤相关指标与PLC的关系及其预后价值尚有待深入研究。

## 6 总结与展望

尽管不同医学中心的研究之间存在明显异质性，且结果不尽相同，甚至存在矛盾，但迄今诸多大规模前瞻性和回顾性临床研究已证实了PLC阳性结果的预后意义。但肺切除前后PLC阳性结果能否成为纳入T分期的指标尚有待学界进一步验证。正如上文所提，对早期肺癌患者而言，PLC阳性结果预后价值更大，且与术后癌性胸膜炎、远期复发密切相关，而使用何种液体进行胸腔冲洗尚无定论。目前在多数医学中心仍使用生理盐水或蒸馏水进行冲洗，而本文所纳入的文献中几乎都使用生理盐水行PLC检测。但冲洗液的用量、液体类别、冲洗时机等可影响研究结果的变量都有待规范。随着近年来早期肺癌的高发，在高通量的外科中心比较不同冲洗液的使用对PLC阳性患者术后结局的影响可对早期患者的治疗效果起到积极意义。

在肺癌根治术中，外科操作不可避免地会造成胸膜破损或淋巴结破碎，这亦可能造成切除术后PLC阳性结果，甚至造成人为的胸膜腔内肿瘤细胞残留而导致术后复发。面对该类情况，目前尚无规范干预措施的共识达成，而常规使用的大量蒸馏水冲洗是否能有效降低切除术后PLC阳性率，以及该种干预能否使患者获得生存获益尚有待多中心前瞻性研究验证。

此外，大量分子生物学研究已证实肿瘤细胞KRAS突变^[39]^、肿瘤分泌的炎症因子^[40]^及肿瘤募集的肥大细胞对恶性胸水形成起到促进作用，相关转化研究及预后靶标在PLC阳性患者中则尚未见报道。因此在胸腔冲洗液中寻找更多与预后密切相关的分子靶标，或可使肺癌术后患者在TNM分期及PLC结果外获得更准确的预后。
